# Pathophysiology of acute lung injury in patients with acute brain injury: the triple-hit hypothesis

**DOI:** 10.1186/s13054-024-04855-w

**Published:** 2024-03-07

**Authors:** Mairi Ziaka, Aristomenis Exadaktylos

**Affiliations:** 1 Clinic for Geriatric Medicine, Center for Geriatric Medicine and Rehabilitation, Kantonsspital Baselland, Bruderholz, Switzerland; 2 Department of Emergency Medicine, Inselspital, University Hospital, University of Bern, Bern, Switzerland

## Abstract

It has been convincingly demonstrated in recent years that isolated acute brain injury (ABI) may cause severe dysfunction of peripheral extracranial organs and systems. Of all potential target organs and systems, the lung appears to be the most vulnerable to damage after ABI. The pathophysiology of the bidirectional brain–lung interactions is multifactorial and involves inflammatory cascades, immune suppression, and dysfunction of the autonomic system. Indeed, the systemic effects of inflammatory mediators in patients with ABI create a systemic inflammatory environment (“first hit”) that makes extracranial organs vulnerable to secondary procedures that enhance inflammation, such as mechanical ventilation (MV), surgery, and infections (“second hit”). Moreover, accumulating evidence supports the knowledge that gut microbiota constitutes a critical superorganism and an organ on its own, potentially modifying various physiological functions of the host. Furthermore, experimental and clinical data suggest the existence of a communication network among the brain, gastrointestinal tract, and its microbiome, which appears to regulate immune responses, gastrointestinal function, brain function, behavior, and stress responses, also named the “gut-microbiome–brain axis.” Additionally, recent research evidence has highlighted a crucial interplay between the intestinal microbiota and the lungs, referred to as the “gut-lung axis,” in which alterations during critical illness could result in bacterial translocation, sustained inflammation, lung injury, and pulmonary fibrosis. In the present work, we aimed to further elucidate the pathophysiology of acute lung injury (ALI) in patients with ABI by attempting to develop the “double-hit” theory, proposing the “triple-hit” hypothesis, focused on the influence of the gut–lung axis on the lung. Particularly, we propose, in addition to sympathetic hyperactivity, blast theory, and double-hit theory, that dysbiosis and intestinal dysfunction in the context of ABI alter the gut–lung axis, resulting in the development or further aggravation of existing ALI, which constitutes the “third hit.”

## Introduction

ABI is a severe and significant health and socioeconomic problem accompanied by high morbidity and mortality [1]. A plethora of clinical and experimental studies demonstrate that ABI represents a complex biochemical cascade associated with numerous pathophysiological processes, which is not limited to the central nervous system affecting the function of multiple distant organs and systems [1, 2]. Among all potential target organs, the lungs appear to be the most vulnerable, with the development of various clinical syndromes, such as ventilator-associated pneumonia [3], adult respiratory distress syndrome (ARDS), and neurogenic pulmonary edema (NPO) [4]. The pathophysiology of the bidirectional brain–lung interactions is multifactorial and involves inflammatory cascades, immune suppression, and dysfunction of the autonomic system [4]. In addition to the “blast theory” and the “pulmonary venule adrenergic hypersensitivity” theories, which combine hydrostatic and high permeability mechanisms resulting in ALI [4], the “double-hit” theory has also been proposed to explain the pathophysiology of ABI-induced ALI as systemic inflammatory response seems to play an integral role in the pathogenesis of pulmonary injury in patients with ABI [5]. The “double-hit” theory refers to the systemic effects of inflammatory mediators in patients with ABI, which create a systemic inflammatory environment (the “first hit”) that makes extracranial organs susceptible to secondary procedures that amplify inflammation, such as mechanical ventilation (MV), surgery, and infections, that is, the “second hit”[5, 6].

The term “gut microbiota” refers to the highly intricate communities of microorganisms inhabiting the intestinal tract, encompassing more than 1000 types of microorganisms representing at least 4000 distinct species [7–10]. These microorganisms engage in a hormonal symbiotic relationship with their host [11]. In recent years, there has been a growing interest in biomedical research due to the recognition that gut microbiota constitutes a critical superorganism and an organ on its own that has the potential to influence various physiological functions of the host [12].

Indeed, emerging evidence over the past few decades suggests the existence of an “invisible” bidirectional communication network among the brain, gastrointestinal (GI) tract, and its microbiome [13]. This communication system appears to govern immune responses, GI function, brain function, behavior, and stress responses [14–18]. Furthermore, beyond these processes, interactions within the gut-microbiome–brain (GMB) axis seem to play a pivotal role in the pathophysiology of various medical conditions, including Alzheimer’s disease, depression, anxiety, inflammatory bowel disease, diabetes, and obesity [18]. ABI is also a pathological entity that negatively impacts the gut microbiome and intestinal function [19].

Until recently, the prevailing belief in the sterility of the lungs [20, 21] hindered systematic exploration of the lung microbiome, resulting in delays in research progress [22]. However, multiple studies have now confirmed that microaspiration or gastroesophageal reflux is common even among healthy, asymptomatic individuals, leading to the colonization of alveoli by microbes [23–26]. The composition of the lung microbiome is closely linked to the host’s immune response and can influence health outcomes, as demonstrated in experimental models and patient cohorts [27]. It is believed that the lung microbiome modulates gene expression in immune cells, leading to the upregulation of various molecules, including interleukin (IL)-5, IL-10, interferon gamma (IFN-*γ*), C–C motif chemokine ligand 11 (CCL11), and promoting toll-like receptor-4 (TLR4)-dependent responses in lung macrophages [28].

In recent years, emerging experimental and epidemiological evidence has highlighted a crucial interplay between the intestinal microbiota and the lungs, called the “gut–lung axis.” Alterations in the composition of the gut microbiome, brought about by factors such as diet, disease, or medical interventions, are associated with modified immune responses and airway homeostasis. The significance of the gut–lung axis has become more apparent with the identification of several gut microbe-derived components and metabolites, including short-chain fatty acids (SCFAs), which serve as key immune system regulators [29]. Indeed, gut dysbiosis can compromise the integrity of the intestinal barrier, potentially leading to bacterial translocation, sustained inflammation, lung injury and, pulmonary fibrosis [30–33].

In the present review, we aim to further elucidate the pathophysiology of ALI in patients with ABI by attempting to develop the “double-hit” theory, proposing the “triple-hit” hypothesis based on recent experimental and clinical data supporting the presence of the gut–lung axis. Specifically, we propose, in addition to sympathetic hyperactivity, blast theory, and double-hit theory, that dysbiosis and intestinal dysfunction in the context of ABI activate the gut–lung axis, leading to the development or further exacerbation of existing ALI, which constitutes the “third hit.”

## Pathophysiology of ALI in patients with ABI

### The blast injury theory

**Fig. 1:**
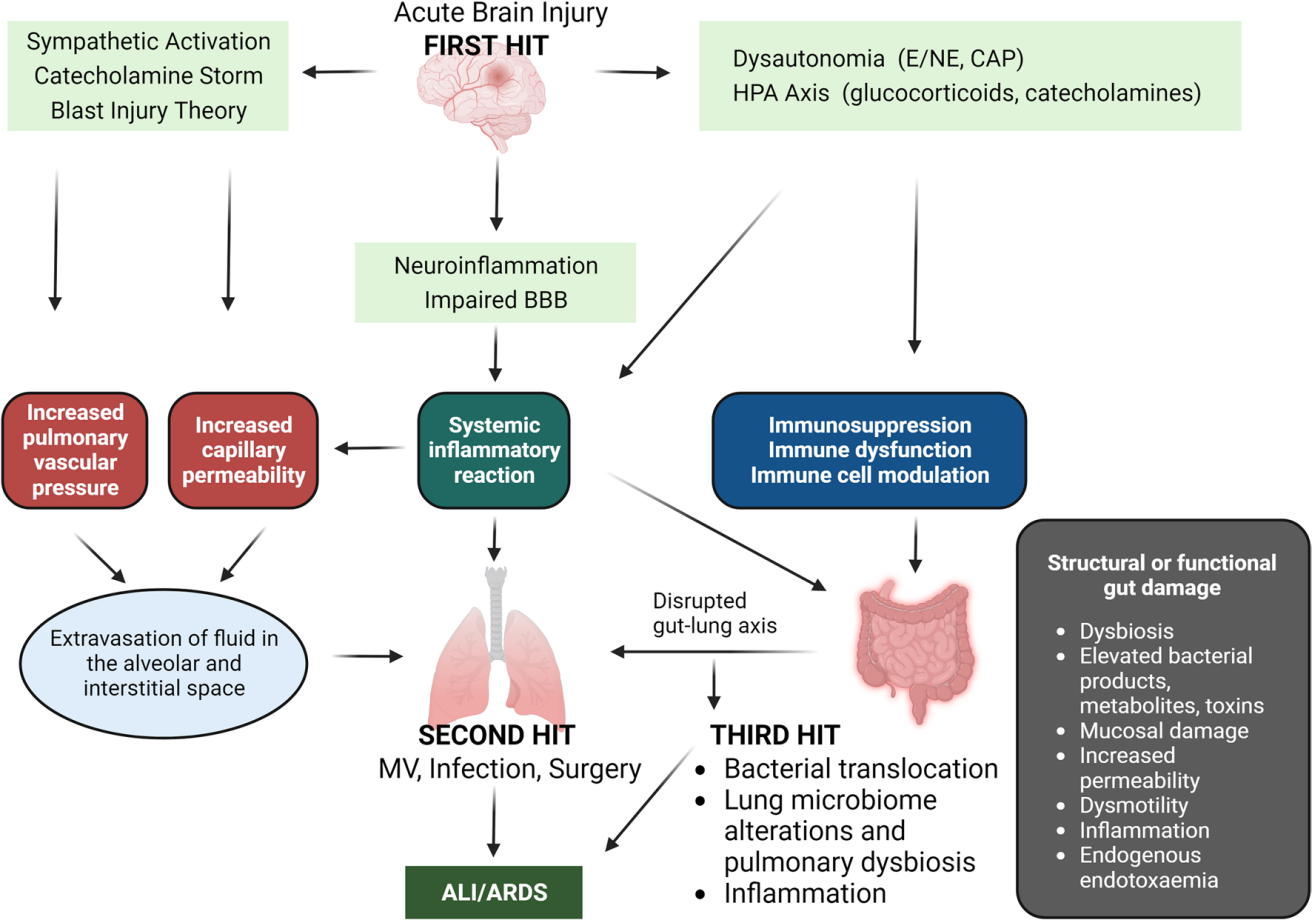
Schematic representation of the “triple-hit” hypothesis. The initial brain injury sets off sympathetic hyperactivity and catecholamine storm, a well-established contributor to ALI. Furthermore, the direct consequences of ABI, characterized by inflammation and oxidative stress (termed the “first hit”), render the lungs susceptible to subsequent interventions like MV, infections, and surgery (referred to as the “second hit”). Notably, the pivotal role of the gut–lung axis in respiratory health reveals that dysbiosis and intestinal dysfunction in ABI patients initiate a sequence of events involving immune dysregulation and microbiome alterations, which subsequently impact the lung tissue. This activation of the gut–lung axis constitutes the “third hit,” culminating in the onset or exacerbation of ALI. ABI: Acute brain injury; ALI: Acute lung injury; ARDS: Acute respiratory distress syndrome; CAP: Cholinergic anti-inflammatory pathway; E/NE: Epinephrine/norepinephrine; HPA: Hypothalamic–pituitary–adrenal; MV: Mechanical ventilation

One of the most proposed theories to explain the pathophysiology of ALI in brain-injured patients is the “blast injury” hypothesis, which proposes that the sympathetic surge following a sudden rise in intracranial pressure triggers a temporary increase in intravascular pressure, leading to the disruption of the alveolo-capillary membrane. The development of NPO can be attributed to either hydrostatic forces, as indicated by a low pulmonary/plasma protein ratio, or high permeability mechanisms, supported by an increased accumulation of protein in the extravascular space of the lungs [34]. The connection between the significant sympathetic discharge and NPO is further substantiated by experimental research, which demonstrates that administering alpha-adrenergic antagonists to brain-injured rats before the onset of injury prevents the hypertensive response and mitigates subsequent lung damage (Fig. 1) [4, 34].

### The double-hit theory

In addition to the “blast theory,” it appears that a systemic inflammatory response plays a crucial role in the development of pulmonary injury in patients with ALI [35]. Indeed, cerebral injury promotes further complex, biochemical, cellular, and metabolic alterations within minutes after the primary event, mainly caused by tissue and cell damage [36], which can persist for even years after the initial injury initiating and maintaining neuroinflammatory and neurodegenerative processes of varying duration leading to a secondary brain injury and damage of distant organs and systems [4, 37]. Both clinical and experimental research in patients and animal models of ABI indicate the existence of an extensive cellular and biochemical cascade initiated within the brain, leading to the production of pro-inflammatory cytokines. Due to the compromised blood–brain barrier (BBB), these cytokines are released into the systemic circulation, triggering inflammatory responses (Fig. 1) [38, 39]. Indeed, similarly to several inflammatory diseases, the dysregulation of BBB has been implicated as central to the pathophysiology of ABI [40, 41], occurs within hours after primary injury, and appears to be biphasic [41–43]. BBB dysfunction may be a direct mechanical consequence of the primary cerebral event or the result of sustained maladaptive inflammatory and cellular processes that contribute to secondary injury [44–46]. The intracranial production of pro-inflammatory cytokines is likely associated with microglia and astrocyte activation [34]. Microglia, the brain’s resident macrophages, undergo morphological and functional activation shortly after ABI [47, 48]. They produce various pro-inflammatory molecules, including IL-1, IL-6, IL-8, and tumor necrosis factor (TNF)-*α* [38]. Experimental studies highlight that in models of moderate diffuse traumatic brain injury (TBI), levels of IL-1*β*, IL-6, and TNF-*α* peak within the cortex 3–9 h after primary injury [49]. These findings are further supported by clinical studies demonstrating increased levels of IL-6, IL-10, IL-8, TNF-*α* and c–c motif chemokine ligand 2 within the first two days post-TBI before gradually return to normal over a period of several weeks [50–52]. This cytokine cataract has been shown to induce astrogliosis and stimulate further microglial activation and axonal dysfunction, indicating an inseparable association between activated immunity and acute ABI [53]. Furthermore, microglial cell activation significantly contributes to BBB disruption, allowing the release of mediators into the systemic circulation [54, 55]. These processes may explain the extracranial organ dysfunctions observed in patients with isolated ABI [46].

Regarding the consequences of neuroinflammation and systemic inflammation in the lungs, Fisher et al. reported elevated cytokine levels in the bronchoalveolar lavage (BAL) of severe BI patients [56]. In addition, a study of lung transplant patients who had elevated IL-8 levels and who received grafts from brain-dead patients experienced graft dysfunction, early recipient mortality, and poor prognosis [57]. Furthermore, a recent study examining BAL within the first 6–8 h after traumatic brain injury and at days 3 and 7 after admission to the intensive care unit (ICU) demonstrated a significant elevation in the concentration of selected apoptotic factors at admission at day 3 and day 7 after the cerebral event [58]. Several experimental studies further corroborate these findings. In animal models with experimentally induced intracerebral hemorrhage, there was considerable neuroinflammation, characterized by marked expression of intracellular adhesion molecule (ICAM)-1 and tissue factor in both the brain and lungs. The authors noted that pulmonary expression of these mediators was associated with structural changes in the lungs [59]. Kalsotra et al. observed significant migration of inflammatory cells into the airways and alveolar spaces 24 h after initiating BI in animal models, accompanied by a substantial increase in pulmonary leukotriene B4 production [60]. Similarly, in an experimental subarachnoid hemorrhage (SAH) model, the lungs exhibited significant expression of ICAM-1, vascular cell adhesion molecule (VCAM)-1, and E-selectin [61].

In summary, the systemic impact of inflammatory mediators in patients with ABI creates a pro-inflammatory environment, and the “first hit” makes extracranial organs susceptible to secondary factors that amplify inflammation, such as mechanical ventilation, which also induces secondary inflammatory reactions, surgery, and infections, constituting the “second hit” (Fig. 1) [5, 62].

## Gut-microbiome–brain interactions

### Gut microbiota

The term gut microbiota refers to the highly complex communities of microorganisms inhabiting the intestinal tract and consists of more than 1000 types of microorganisms from at least 4000 distinct species [7–10] while establishing a hormonal symbiosis with their host [11]. The human GI tract of healthy individuals is inhabited by commensal microbes of all three life domains, that is, bacteria, archaea, viruses, and eukarya, whereas the bacterial representation constitutes the dominant one [63, 64]. The gut microbiota composition appears to show variations depending on the age and genetic factors and parameters, which are modifiable as diet and lifestyle, external stressors, medications including non-steroidal anti-inflammatory drugs, and antibiotics, illness, and sepsis [64–68].

In recent years, there has been an increasing interest in biomedical research, given that gut microbiota represent a crucial superorganism and an organ itself that could potentially modify various physiological functions of the host [12]. Indeed, during the last decades, emerging evidence suggests that there is an “invisible” cross talk between the brain, gastrointestinal tract, and its microbiome [13], which appears to regulate immune responses, GI tract function, brain function, behavior, and stress responses [14–18]. In addition to the processes above, GMB interactions seem to play a pivotal role in the pathophysiology of a variety of pathological conditions, for example, ABI, such as TBI, Alzheimer’s disease, depression, anxiety, inflammatory bowel disease, diabetes, and obesity [18].

### Gut microbiome in patients with ABI

The bidirectional communication pathway between the central nervous system and the gut involves the central and enteric nervous systems [7, 69], the hypothalamic–pituitary–adrenal axis (HPA), and immunological pathways (Fig. 1) [70]. The autonomous nervous system is a key communication instrument between the brain and the gut, regulating intestinal homeostasis, gut mobility and permeability, bile secretion, fluid maintenance, mucosal neuroimmune response, and immune cell activation [70, 71].

The pathophysiological consequences of ABI have garnered growing attention in the context of intestinal dysfunction. This communication encompasses various types of signals and pathways, including neuronal, hormonal, and immunological cross talk, and involves both afferent and efferent signals [18]. Indeed, experimental studies in brain and spinal cord injured animal models have convincingly demonstrated that central nervous system injury affects the motility and permeability of the intestinal wall [72, 73] and modifies the composition of the gut microbiome [70, 74], resulting to gut dysbiosis [75]. Numerous investigations have demonstrated that following moderate-to-severe TBI in rodent models, there is an acute surge in mucosal damage and increased permeability within the small intestine, typically occurring within the first 72 h post-injury [72, 76]. Moreover, it has been shown that in the context of TBI, the enterocyte population and its barrier function are severely compromised, resulting in the disruption of tight junctions and an elevation in enterocyte permeability. In the absence of luminal nutrients, gastrointestinal motility is diminished, fostering an environment conducive to bacterial overgrowth. These bacteria are more prone to adhere to enterocytes, provoking apoptosis in these cells and consequently amplifying enterocyte permeability [72]. Recently, Mahajan et al. conducted a similar study, investigating samples for microbial growth from rectal swabs obtained on days 0, 3, and 7 after admission in patients with moderate–severe TBI and found widespread colonization with *Proteobacteria phylum* with *Enterobacteriaceae* forming the largest group [77]. Experimental studies further support these findings. Houlden et al. have shown that ABI in the context of experimental stroke influences bacterial communities in the caecum and that brain injury is associated with specific changes in gut microbiota [70]. Moreover, in a mouse model—known as controlled cortical impact (CCI) and designed to simulate TBI—significant alterations in the gut microbiota were observed. Specifically, there was a notable decrease in *Lactobacillus gasseri*, *Ruminococcus flavefaciens*, and *Eubacterium ventriosum*, accompanied by a substantial increase in *Eubacterium sulci* and *Marvinbryantia formatexigens* at 24 h post-CCI [78]. In addition, clinical studies in patients with stroke highlight significant gut dysbiosis associated with intestinal dysfunction and intestinal bleeding complications, as well as septicemia of intestinal origin, which has a negative impact on the prognosis [79].

### Impact of sympathetic hyperstimulation in GMB axis

Accumulating data highlight that neurotransmitters contribute significantly to GI physiology. In recent years, epinephrine, norepinephrine, dopamine, and serotonin have been the subject of intensive research due to their influence on gut function and their potential participation in gastrointestinal and central nervous system pathophysiology. It has been shown that neurotransmitters ultimately impact gut motility, nutrient absorption, GI innate immune system, and the microbiome (Fig. 1) [80]. Furthermore, catecholamines interface with adrenergic receptors located on the cell membranes of visceral organs and smooth muscles. This interaction triggers the activation of signaling cascades, subsequently inducing modifications in organ function and smooth muscle tone [81]. These observations are further supported by experimental studies demonstrating that autonomic dysregulation and increased sympathetic tone may modify gut permeability in experimental stroke models [82]. Furthermore, it is well documented that the postganglionic sympathetic neurons are of neuralgic importance for the function of the GI system’s lymphatic tissue and immune cells and that adrenergic receptors are expressed by the cells of the innate immune system [83]. Therefore, in case of marked catecholamine secretion, such as in patients with ABI [4], their binding to adrenergic receptors in the gastrointestinal tract could modify inflammatory processes at the local level [84] partly by modifying the migration and function of immune cells, thereby stimulating the production and release of inflammatory mediators [84–86]. Furthermore, previous studies have demonstrated the supportive effect of noradrenaline and adrenaline via *α* and *β* adrenergic receptors on the growth of gram-negative microbes [87]. In addition, it appears that the presence of catecholamines enhances the growth of *Helicobacter pylori* and *Campylobacter jejuni* [88, 89].

Consistent with the sympathetic hyperstimulation observed in patients with ABI [4], an experimental study was able to demonstrate gut dysbiosis and intestinal dysfunction mediated by dysregulated sympathetic nerve signaling within the submucosal plexus of the gut following stroke [82]. These findings are further supported by the observation that pharmacological *β*-adrenergic receptor blockade with propranolol or metoprolol reestablishes gut permeability in stroke-affected mice to a degree similar to sham-operated mice [82]. In accordance with these findings, a further experimental study in a mouse model of stroke highlighted that noradrenaline excess after stroke results in an imbalance of the host microbiota, mucoprotein, and goblet cell numbers in the cecal [70].

### Impact of inflammation on the GMB axis in patients with ABI

As mentioned above, ABI-induced systemic inflammation has been associated with the impairment of peripheral organs and systems, including the gut [56, 90, 91]. Modifications of the physiological interactions between the brain and the enteric and central nervous systems have been implicated in the pathophysiology of peripheral organ damage after TBI [92]. Furthermore, ABI-mediated sympathetic neural stimulation results in the release of inflammatory cytokines in peripheral organs, including the gut [93, 94]. In critically ill trauma patients, the intestinal inflammatory reaction is mediated by the recruitment of neutrophils and monocytes into the intestinal tissue, which in turn results in the release of inflammatory cytokines and superoxide molecules, which have the capability to induce damage to the intestinal mucosa. Studies in patients with TBI have underscored the significance of the localized intestinal inflammatory response, which is mediated by an increase in the concentration of intestinal cytokines, including TNF-*α*, which potentially can modify the structure and function of tightly bound proteins in the intestinal epithelium [95]. Moreover, experimental studies investigating the effects of the introduction of fecal content from individuals who have experienced a stroke into germ-free mice were associated with meaningful outcomes regarding stroke. This aggravation occurred due to the initiation of a pro-inflammatory immune response mediated by T-helpers (Th) 1 and Th17 cells [96], indicating that stroke-associated dysbiosis mediates an immune response characterized by pro-inflammatory processes, ultimately exacerbating brain damage in the context of stroke. In addition, a recent study investigating the gut microbiota and inflammatory mediators of patients with post-stroke cognitive impairment (PSCI) showed that PSCI patients had significantly higher levels of gut *Enterobacteriaceae*, lipopolysaccharide (LPS) and peripheral inflammation markers. Moreover, fecal microbiota transplantation from PSCI patients to stroke mice was associated with a higher level of *Enterobacteriaceae*, intestinal Toll-like receptor-4 (TLR4) expression, circulating LPS, LPS-binding protein and inflammatory cytokines, and a decreased level of fecal butyrate, more intense intestinal damage and cognitive impairment than mice that received microbiota from non-PSCI patients (non-PSCI mice) [97]. Similarly, Roy Sarkar et al. have pointed out that disturbances in the intestinal microbiome can lead to elevated levels of LPS, pro-inflammatory cytokines, Th cells, and monocytes [98]. Furthermore, it is worth noting that ischemic stroke patients who experience post-stroke depression (PSD) exhibit dysregulated intestinal flora and insufficient growth of *Bifidobacterium*. Conversely, *Enterococcus faecalis* and *Escherichia coli* levels are markedly elevated in these patients, and these increases are positively correlated with the levels of interleukin IL-1, IL-2, IL-6, and C-reactive protein in their serum. On the contrary, the content of *Bifidobacterium* showed a negative association with IL-1 and IL-2 levels in their serum [99].

## Gut–lung interactions

### Lung microbiome

Until recently, the widely held belief of the sterility of the lungs [20, 21] hindered the systematic exploration of the lung microbiome, resulting in delays in research progress [22]. However, multiple studies have now confirmed that microaspiration or gastroesophageal reflux is common even among healthy, asymptomatic individuals, leading to the colonization of alveoli by microbes [23–26]. Culture-independent molecular techniques have revealed a diverse bacterial community in the lower airways of healthy individuals, primarily comprising *Prevotella*, *Veillonella*, *Streptococcus*, and *Fussobacterium species* [100–102]. Nevertheless, the microbial biomass in healthy lungs remains low (10^3^ to 10^5^ bacteria per gram of tissue) [102–104], primarily due to limited nutrient availability.

The lung microbiome’s composition is closely tied to the host’s immune response and can impact health outcomes, as demonstrated in experimental models and patient cohorts [27]. Evidence from human data and experimental models suggests that the intensity of microbial clearance varies and is related to the specific aspirated microbial species. Even when oral commensals are promptly cleared from the lower airway, such events lead to persistent and dynamic alterations in the lower airway’s immune environment [105]. Experimental models have emphasized the critical role of early-life airway microbiota in shaping a functional immune system, influencing Helios (−) regulatory T cells (Tregs), and reducing susceptibility to allergic respiratory conditions [106]. Furthermore, the early establishment of the microbiome in the airways affects the upper respiratory tract’s microflora, which in turn impacts susceptibility to infectious diseases [107].

Numerous studies consistently show a strong correlation between the upper and lower respiratory tract microbiomes, particularly in cases of acute and chronic inflammation [108–111]. Microaspiration primarily drives this association, especially in cases involving gastroesophageal reflux disease or impaired airway cleansing mechanisms [108]. The respiratory microflora plays a crucial role in promoting the differentiation of peripheral Tregs, vital for regulating type 2 immune responses. Experimental research also highlights an intricate interplay between the microbiome and natural killer T cells (NKT cells) [112, 113]. Moreover, studies employing models lacking airway microflora have demonstrated increased eosinophils and Th2-lymphocytes in the lungs of animals [112, 113]. The lung microbiome is believed to modulate gene expression in immune cells, influencing the upregulation of various molecules, including IL-5, IL-10, IFN-*γ*, CCL11, and TLR4-dependent responses in lung macrophages [28].

### Gut–lung interactions in critically ill patients with ABI

ABI is a global concern associated with elevated morbidity and mortality rates among adults [4, 114–116]. Indeed, nearly 50% of hospitalized TBI patients require intensive care due to secondary brain injury risks [117]. These patients are prone to infections, necessitating mechanical ventilation [118], with approximately 40% receiving prophylactic antibiotics [119]. Moreover, almost 70% of moderate-to-severe TBI patients develop early multi-organ dysfunction [120].

Critical illness and injury diminish microbial diversity, alter bacterial communities, and promote opportunistic microorganisms [121, 122]. The gut microbiome significantly influences distant organs, including the brain, liver, skin, and heart [123], as well as respiratory diseases [124, 125]. The gut–lung axis connects the gut microbiota to lung inflammation, impacting local and systemic immune responses [26, 126, 127] through the release of metabolites and endotoxins [128, 129]. Dysbiosis in the gut microbiota can contribute to respiratory diseases like asthma and COPD by disrupting immune, hormonal, and metabolic homeostasis [26, 130–133]. The reason why patients with intestinal pathologies are prone to pulmonary inflammation and lung diseases remains uncertain. This could be attributed to the common embryologic origin resulting in inherent similarities between these organs [134, 135].

The gut–lung axis operates through direct and indirect pathways involving substances like peptidoglycan and LPS activating the host’s immune response, SCFA affecting immune cell development, immune cells migrating between the gut and lungs via the bloodstream, and microbial metabolites influencing the host’s type I interferon response [136].

Dysbiosis can compromise the intestinal barrier, potentially leading to bacterial translocation, sustained inflammation, lung injury, and pulmonary fibrosis [31–33, 97]. Gut barrier disruption is prevalent in critically ill patients, as the dense mucus layer may be compromised [137, 138]. Lung microbiome disruptions can also result from gut–lung cross talk in critically ill patients [139, 140], with a shift toward gut-associated bacteria in the lungs [140]. Bacterial translocation may occur via gut-draining lymphatics, the portal system, or systemic circulation (Fig. 1) [140–143].

Increased gut permeability, or “leaky gut,” is common in critically ill patients [144, 145], exacerbated by factors like inflammatory mediators (IFN-*γ*, IL-6, TNF-*α*) disrupting tight junctions and intestinal hypoperfusion during stress reactions [145]. Anaerobic bacteria imbalances can further harm the gut immune barrier, promoting pathogenic bacteria growth and the production of inflammatory mediators [97, 146, 147]. In critical illness, luminal components from the small intestine, including bacteria, LPS, and pro-inflammatory molecules, can reach the lungs via the portal circulation or mesenteric lymph vessels, promoting alveolar inflammation and acute lung injury [126, 143, 148].

Moreover, elevated concentrations of both endogenous and exogenous catecholamines have been shown to influence the lung bacterial population [149, 150]. Indeed, previous research has ultimately highlighted an in vitro correlation between catecholamines and the growth of select bacteria, including *Pseudomonas aeruginosa (Ps. aeruginosa)* and *Streptococcus pneumonia,* which are classic bacterial representatives of the lung microbiome [87, 151, 152]. Human bronchoalveolar lavage samples have provided evidence that heightened alveolar catecholamine levels are strongly associated with the dominance of a single bacterial species, most frequently *Ps. aeruginosa* [153]. These findings are consistent with those of an in vitro study by Freestone et al. investigating the association of catecholamines with the growth and virulence of *Ps. aeruginosa*. The authors reported a strong correlation between catecholamines and *Ps. aeruginosa* growth, as reflected by 50-fold increases in bacterial numbers. The underlying pathophysiological mechanisms involve inotrope-associated delivery of transferrin-iron, internalization of the inotrope, and upregulation of the key pseudomonal siderophore pyoverdine [151]. It has been suggested that the lung is susceptible to elevated concentrations of neurochemicals associated with stress due to its ample blood supply and dense noradrenergic tissue innervations [87]. Therefore, any source of alveolar injury and inflammation, whether direct—such as aspiration or ventilator-induced lung injury—or indirect—such as sepsis or shock, can initiate a sequence of inflammatory events resulting in elevated intra-alveolar catecholamine concentrations, which, in turn, foster the growth and virulence of specific bacterial community members and contribute to a disrupted bacterial community that sustains alveolar inflammation [154, 155]. Remarkably, Kopin et al. calculated that in non-septic situations, the concentration of norepinephrine at the receptor site is at least three times greater than what can be measured in the bloodstream. Consequently, utilizing plasma measurements to ascertain catecholamine levels during an acute condition reflects a spill-over effect that significantly underestimates the actual catecholamine levels within the organ site [156, 157]. Additionally, bacterial growth promotion is not limited to catecholamines; it is also seen with inflammatory mediators like TNF-*α*, IL-1, IL-6, and IL-8, as well as glucocorticoids, molecules that are excessively produced in ABI [4, 158–161].

As previously mentioned, ABI triggers a robust inflammatory response within the brain, which is accompanied by the suppression of the peripheral immune system, a phenomenon referred to as ABI-induced immunosuppression [4]. Some key factors contributing to ABI-induced immunosuppression include the shift of lymphocytes from a Th1 phenotype to a Th2 phenotype, reductions in lymphocytes and NKT cells in both blood and the spleen, as well as impairments in the defensive functions of monocytes and neutrophils [162]. Moreover, one potential factor contributing to ABI-induced immunodeficiency is the activation of the sympathetic nervous system and the HPA axis by the cerebral event [4, 163]. An experimental study in a stroke model further supports this notion, demonstrating that the injection of 200 colony-forming units of *Streptococcus pneumoniae* into the nasal cavity of stroke-afflicted mice can lead to the development of pneumonia and bacteremia, whereas 200,000 colony-forming units are required in control animals to induce similar conditions. [164]. To complicate things further, experimental research indicates that alterations in gut microbiome have detrimental consequences on pulmonary defense against pathogens. Indeed, an experimental model of antibiotic-treated and germ-free mice showed an enhanced vulnerability to lung infections with *Streptococcus pneumoniae* and *Klebsiella pneumoniae* due to low pulmonary levels of IL-17 and granulocyte–macrophage colony-stimulating factor (GM-CSF) in these mice, which are required for an effective lung defense [165].

### Lung microbiome in patients with acute lung injury

As mentioned above, critical illness is associated with alterations of the lung microbiome [100, 139, 166, 167], which are significantly related to systemic and local inflammation [139, 166]. Bacterial diversity undergoes a reduction, and commensal microbial populations may be displaced by potential pathogens, often stemming from alternative ecosystems, including the GI tract and the skin [100, 168]. The crucial cross talk between the intestinal microbiota and the lungs receives notable clinical importance as this microbiome shift is associated with remarkable inflammatory processes and lung injury [139, 166, 169]. Specifically, the lung microbiome of patients with ALI is fortified with gut-associated bacteria (e.g., species of the *Enterobacteriaceae* family [139, 140]), which is associated with the subsequent development of ARDS [139]. Indeed, while the enrichment of lung microbiome with gut-originated bacteria could reflect a generalized state of dysbiosis in these severely injured patients, the study by Panzer et al. indicates a drastic participation of these microbes in the pathophysiology of ARDS [139]. The authors highlight that in critically ill mechanically ventilated patients, lung dysbiosis at the early stages of critical illness is associated with a remarkable increase of inflammatory mediators (IL-6, IL-8), which in turn predisposes these patients to the subsequent development of ARDS [139]. These findings are in accordance with the findings of Dickson et al., who investigated the lung bacterial composition in BAL specimens from ARDS and non-ARDS patients. The authors reported *Streptococcaceae*, *Veillonellaceae*, *Prevotellaceae*, *Verrucomicrobiaceae*, and *Flavobacteriaceae* as the dominant species in patients without ARDS, whereas *Pasteurellaceae* and *Enterobacteriaceae* are identified in BAL specimens of ARDS patients [170]. Furthermore, an experimental study of lung injury following abdominal sepsis induced by cecal ligation and puncture found that the lung microbiome of rodents was enriched with gut bacteria [171]. These findings are further supported by the study of Schmitt et al., who compared the composition of the lung microbiome in 15 patients with sepsis-induced ARDS undergoing abdominal surgery and 15 patients post-esophagectomy. The authors observed a decreased *α*-diversity index of the pulmonary microbiome in ARDS patients, which was related to the length of the ICU stay and the need for ventilator support, indicating that an imbalance of the lung microbiome may contribute to the pathogenesis of ALI/ARDS in patients with sepsis [172]. The data, however, in patients with ABI are scarce. A recent study investigating the lung microbiome of patients with TBI receiving specialized (16pts) or standard nutrition reported that the BAL microbiota of patients who develop VAP showed significant differences in beta diversity and high composition of *Staphylococcus* and *Acinetobacter* Genera [173]*.*

In contrast to the GI tract, the alveolar space is characterized by an ecologically unfavorable milieu for the majority of bacteria, resulting in minimal bacterial reproduction [166]. A significant factor contributing to this low reproductive capacity is the scarcity of nutrient substrates essential for bacterial metabolism. Unlike the nutrient-rich environment of the gut lumen, the alveolar space is relatively barren, containing only a thin layer of lipid-rich surfactant along the epithelial lining. From the bacterial perspective, healthy alveoli can be considered inhospitable. However, in conditions characterized by alveolar injury, such as ARDS or pneumonia, the environmental conditions undergo a dramatic shift [153, 166, 174, 175]. The pathophysiology of the development of microbial dysbiosis in the lungs of critically ill patients is multifactorial and includes alterations of local physiochemical and metabolic local characteristics such as pH, oxygen concentration, occurrence of free radicals, and nutrients within the alveoli [20, 175, 176]. The emergence of anaerobic zones, attributable to alveolar edema or alveolar collapse and thus atelectasis in injured lungs, creates a more conducive environment for the proliferation of potential pathogens [20]. Furthermore, the presence of an endotracheal tube in mechanically ventilated patients facilitates continuous microaspiration of oropharyngeal flora while impeding the natural clearance mechanisms of the airways [177]. The alveolar airspaces, which were previously devoid of content, become inundated with fluid rich in proteins. This phenomenon is particularly evident in patients with NPO, representing a newfound and ample energy source for proliferating microorganisms [4, 175]. Notably, NPO is defined as the extravasation of protein-rich fluid into the interstitial and alveolar spaces of the lungs following various central nervous system pathologies, including stroke, SAH, subdural hemorrhage, status epilepticus, central nervous system infections, and TBI [178–181]. Moreover, the bactericidal surfactant layer becomes deactivated, and the process of eliminating microbes is hindered due to impaired mucociliary clearance. From an ecological standpoint, the alveoli, when injured, start to exhibit similarities to the gut environment rather than resembling healthy lung tissue. Consequently, it is not unexpected that a majority of pathogens that emerge during critical illness have their origins in the GI tract. The interplay between the microbiome and alveolar injury can drive a dysregulated feedback loop that extends across the host-microbiome interface [166].

## The “triple-hit” hypothesis

The "triple-hit" hypothesis is grounded in the idea that ABI patients experience a threefold impact on their lung pathophysiology. The initial brain injury triggers sympathetic hyperactivity, which has long been recognized as a contributor to ALI. Moreover, the direct effects of ABI, including inflammation and oxidative stress (“first hit”), make the lungs vulnerable to secondary procedures such as mechanical ventilation (“second hit”) [4]. Finally, based on recent research which has uncovered the significance of the gut–lung axis in respiratory health, dysbiosis, and intestinal dysfunction in ABI patients initiates a cascade of events involving immune dysregulation and microbiome alterations, which subsequently affect the lung tissue [21, 166, 170]. This activation of the gut–lung axis forms the “third hit,” leading to the development or worsening of ALI (Fig. 1). Our hypothesis could be used as a theoretical background for research on prophylactic or therapeutic strategies, which will target the GMB axis in order to mitigate the risk for ALI that is exacerbated by systemic inflammation, immunosuppression, and bacterial translocation in this vulnerable patient’s population.

## Therapeutic implications

### Mechanical ventilation in brain-injured patients with acute lung injury

Managing patients with ALI and severe ABI poses a considerable therapeutic challenge. Traditional approaches to treating ARDS may conflict with the management of elevated intracranial pressure (ICP) and reduced cerebral perfusion pressure (CPP) [182]. The lack of inclusion of brain-injured patients in most clinical trials investigating MV in ARDS has resulted to the lack of substantial and reliable data to instruct clinicians in the management of ARDS in this specific population [4, 183]. Protective MV has demonstrated improved survival in ARDS [183], but it could lead to hypercapnia and hypoxemia, potentially contributing to increased ICP and cerebral hypoxia [184]. For neurocritical care patients, maintaining normal partial pressures of arterial oxygen (PaO_2_) and preventing hypoxia are of paramount importance. An analysis of the IMPACT study database, encompassing over 9000 TBI patients enrolled in randomized controlled trials and series since the 1980s, has confirmed the impact of hypoxemia on mortality and unfavorable outcomes [185]. A guideline from the European Society of Intensive Care Medicine recommends maintaining PaO_2_ levels between 80 and 120 mmHg in brain-injured patients [186].

Protective MV has been shown to enhance survival in ARDS [183]. However, it may result in hypercapnia and hypoxemia, potentially contributing to increased ICP and cerebral hypoxia [184]. In ABI patients, the maintenance of sufficient oxygenation and the prevention of hypoxia are of paramount significance. Analyzing the IMPACT study database, which comprises over 9000 patients with TBI enrolled in randomized controlled trials and series dating back to the 1980s, has confirmed the impact of hypoxemia on mortality and unfavorable outcomes [185]. Indeed, recent recommendations regarding the ventilation targets in brain-injured patients with ARDS suggest maintaining PaO_2_ levels between 80 and 120 mmHg in brain-injured patients [186]. This recommendation contrasts with the ARDSnet practice, which sets oxygenation goals between 55 and 80 mmHg [183], allowing a relatively mild hypoxia. Nevertheless, given that patients with severe ABI face up to a 50% higher likelihood of death when the PaO_2_ is below 110 mm Hg [187–189], it might be justifiable to adopt a strategy targeting a higher PaO_2_ than the one recommended for ARDS patients without ABI [182, 190]. Indeed, if direct measures of brain physiology, including invasive intracranial pressure and brain tissue oxygen monitoring, are available, an individualized target of PaO_2_ is strongly recommended. In cases where direct measurement is unavailable, aiming for PaO_2_ > 110 mm Hg is suggested, considering lung compliance limitations [182]. However, it is crucial to take into account that both hypoxemia and excessive hyperoxemia have been linked to elevated mortality and a reduction in favorable outcomes among patients with ABI [188].

Lung-protective ventilation may induce hypercapnia, which is acceptable as long as oxygenation is secured and the pH level remains above 7.1 [191, 192]. Nevertheless, hypercapnia can negatively impact cerebral hemodynamics and the autoregulation of cerebral blood flow, worsening a preexisting impairment of cerebral autoregulation in neurocritical care patients [193–196]. Indeed, the cerebral vasculature exhibits high responsiveness to carbon dioxide (CO_2_) levels, and hypercapnia may lead to vasodilation of cerebral arterioles with subsequent intracranial hypertension and brain tissue hypoxia, which is poorly tolerated after ABI [184]. Nonetheless, a recent meta-analysis indicates that low tidal volumes lead to a modest increase in partial pressure of arterial carbon dioxide (PaCO_2)_, shifting from 38 to 41 mmHg [197]. Based on brain physiology parameters, a tailored strategy targeting normal PaCO_2_ values (35–45 mm Hg) could be considered [182] by adjusting respiratory rate instead of tidal volume, given that a low tidal volume (7.0 ml/kg of ideal body weight) results in minimal alterations of the PaCO_2_ level [198].

Positive end-expiratory pressure (PEEP) hinders alveolar collapse, preserving oxygenation, enhancing functional residual capacity, and optimizing ventilation-perfusion matching [199–201]. However, the application of PEEP potentially elevates ICP by increasing intrathoracic pressure, thereby compromising venous return from the brain [202]. Moreover, the application of PEEP could induce compensatory vasodilation, potentially leading to intracranial hypertension under conditions of impaired cerebral autoregulation and reduced intracranial compliance. In addition, when cerebral autoregulation is compromised, an elevation in PEEP might decrease CPP, resulting in cerebral ischemia [4]. Concerns regarding elevated ICP lead to the delivery of PEEP at levels not exceeding 5 cm H_2_O in up to 80% of patients with brain injury. Nevertheless, a retrospective study evaluating the impact of PEEP on intracranial pressure in 20 patients with ABI complicated by ALI highlighted that across the range of 0 to 15 cm H_2_O, variations in PEEP levels showed no significant influence on ICP or CPP. Moreover, the effect of PEEP on ICP is likely minimal when PaCO_2_ is adequately controlled [202]. Currently, applying PEEP for ARDS treatment in ABI patients is considered appropriate, with the necessary condition that mean arterial pressure (MAP) is upheld. A consensus panel recommendation suggests using the same PEEP levels for brain-injured patients as those without brain injury unless elevations in ICP are associated with increased PEEP. Monitoring ICP in ABI patients with ALI can aid in the titration of PEEP [186]. However, recruitment maneuvers with high PEEP levels should be avoided as they have the potential to significantly elevate ICP in patients with compromised cerebral autoregulation due to the impairment of jugular blood outflow, elevated intrathoracic pressure, increased central venous pressure (CVP), and impediment of cerebral venous return to the right atrium [203].

Finally, other therapeutic options, including pulmonary vasodilator therapy, steroids, prone positioning, supine chest compression, and extracorporeal membrane oxygenation, might individually be considered [182].

### Targeting the brain–gut–lung axis

As mentioned above, extensive research has demonstrated that bacterial metabolites derived from the intestine, for example, SCFAs, exert influence on local and systemic immune responses, regulate the maturation and function of microglia, and modulate the integrity of both the gut and the BBB [204]. Notably, SCFAs have the capacity to traverse the BBB and enhance its integrity, playing a pivotal role in brain homeostasis [215, 216] and might potentially contribute to the limitation of ABI-associated inflammatory injury of peripheral organs, including the gut and the lung. Additionally, SCFAs participate in a crucial way in the immunomodulatory properties of probiotics and prebiotics. Probiotics and prebiotics could modify significantly the pathogenesis of inflammatory processes by modulating the intestinal microbiota. This modulation involves the enhanced proliferation of health-beneficial microorganisms and the decreased presence of pathogenic microorganisms [205]. The impact of probiotics and prebiotics on the development and maturation of innate and adaptive immunity occurs via the secretion of cytokines, including IL-10 and transforming growth factor-*β* (TGF-*β*). As stated previously, these cytokines play a pivotal role in controlling the development of Treg cells and influencing the response of Th cells (Th1/Th2). This regulatory process extends to the release of TNF-*α*, interferons, and chemokines by immune cells [206]. Moreover, clinical studies have shown that the administration of probiotics in patients with ABI results in a decrease in infection rates through the reduction of IL-4 and IL-10 levels and the balance of Th1 and Th2 cytokines. Additionally, the use of probiotics was associated with a decrease in ICU stay and the number of days requiring mechanical ventilation [207, 208].

Fecal microbiota transplantation, characterized by the infusion of feces from a healthy individual into a patient with disrupted gut dysbiosis, has shown therapeutic applications in cases of ABI [209–212]. This therapeutic approach has been identified as effective in restoring gut microbiota dysbiosis following ABI, demonstrating an additional neuroprotective effect [96, 213]. Ultimately, treatment modalities, including vagus nerve stimulation and administration of gut-derived neuropeptides such as ghrelin, which can potentially modify the inflammatory response seen in patients with ABI, both systemically and locally [74], microbial engineering approaches and bacterial gene therapy significantly improve peripheral organ damage by addressing the dysbiosis of gut microbiota induced by acute CNS injury [214].

## Conclusion

The “triple-hit” hypothesis extends our current understanding of the pathogenesis of ALI in ABI patients by incorporating the emerging concept of the gut–lung axis. By considering the interplay of sympathetic hyperactivity, the blast theory, inflammatory cascades, and gut-related pathogenetic mechanisms, this hypothesis provides a more comprehensive framework for investigating and understanding ALI in the context of ABI. Further research in this direction promises to shed light on novel therapeutic strategies that may improve the prognosis of these vulnerable patients.

## Data Availability

Not applicable.
